# Incarcerated Thoracic Gastric Herniation after Nephrectomy: A Report of Two Cases

**DOI:** 10.1155/2013/896452

**Published:** 2013-05-16

**Authors:** Conall Fitzgerald, Orla Mc Cormack, Faisal Awan, Jessie Elliott, Narayanasamy Ravi, John V. Reynolds

**Affiliations:** ^1^Department of Surgery, St. James's Hospital, Dublin 8, Ireland; ^2^Trinity Centre, St. James's Hospital, Dublin 8, Ireland

## Abstract

Iatrogenic diaphragmatic hernias can occur after abdominal or thoracic surgery. Acute presentation of a diaphragmatic hernia varies depending on the extent and nature of the organ which has herniated. The initial diagnosis can be challenging due to the nonspecific nature of the presenting symptoms. Delay in diagnosis poses a significant risk to the patient, and a rapid deterioration can occur in the context of strangulation. We outline two cases of acute gastric herniation through a defect in the diaphragm after an open and a laparoscopic nephrectomy. Both had characteristic findings on imaging, required emergency, surgery and had a successful outcome. Both cases highlight the potential for late presentation with non-specific symptoms and the necessity for urgent surgical management where gastric perfusion is compromised.

## 1. Introduction

Diaphragmatic hernias are classified as congenital or acquired, congenital hernia types being Bochdalek (95% of cases), Morgagni (2% of cases) and due to diaphragm eventration or central tendon defects. Acquired diaphragmatic hernias are hiatal, traumatic either due to blunt force or penetrating injuries, or iatrogenic. Iatrogenic diaphragmatic hernias are rare complications of thoracic or abdominal surgery, having been described following oesophagectomy, gastrectomy, laparoscopic cholecystectomy, fundoplication, gastric banding, radiofrequency ablation of liver lesions, thoracotomy, splenectomy, and nephrectomy [1–12]. An acute presentation with a strangulated or an obstructed viscus may present a diagnostic dilemma and require urgent resection and repair. 

We describe herein two cases of delayed diaphragmatic hernia following nephrectomy which resulted in gastric incarceration requiring emergency repair. Both cases highlight late presentations, the potential for late diagnosis, and classical radiological features where high quality computed tomography (CT) is utilized, as well as the need for urgent surgery once the diagnosis is established. 

## 2. Case One

A 35-year-old male with polycystic liver and kidney disease, on dialysis, underwent an open bilateral nephrectomy three years prior to a presentation to his local hospital with chest pain, dyspnoea, and a low-grade fever. He was treated for suspected pneumonia, but three days later his condition deteriorated and he progressed rapidly into septic shock with resultant intubation and mechanical ventilation and requirement for inotropic support. 

**Figure 1:**
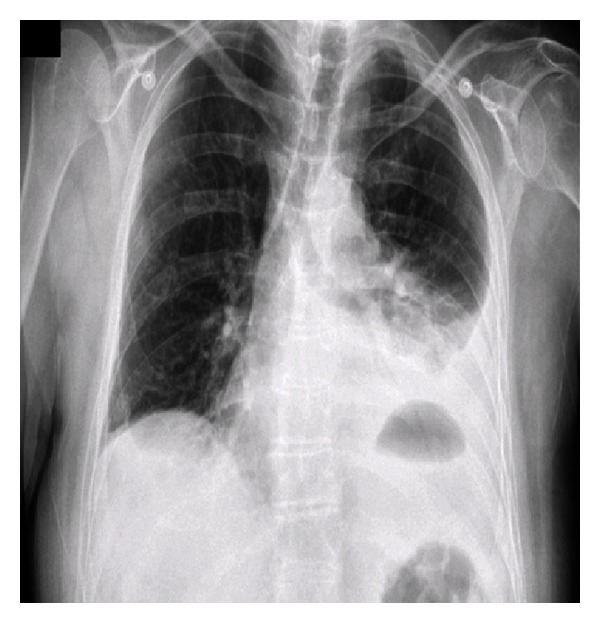
CXR Case 1; left-sided pleural effusion with a thoracic air-fluid level and slight mediastinal shift.

**Figure 2:**
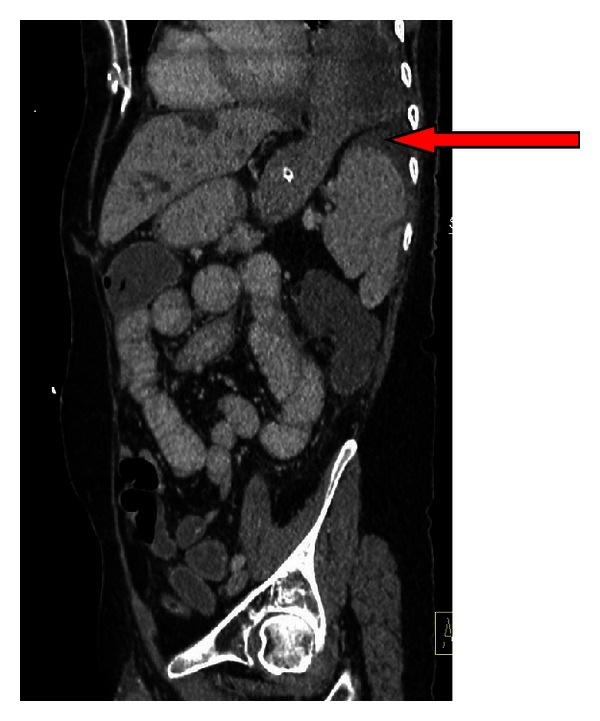
CT Case 1; saggittal section showing herniation of the gastric fundus through a diaphragmatic defect (as indicated by arrow) with evidence of incarceration-note nasogastric decompression of the abdominal portion of the stomach in the presence of a dilated nondecompressed thoracic stomach.

A chest radiograph (CXR) at that time (Figure 1) revealed a left-sided pleural effusion with a thoracic air-fluid level and slight mediastinal shift. He was transferred to the Oesophageal and Gastric Centre at St James's Hospital in Dublin, where a CT thorax and abdomen clearly established the diagnosis of herniation of the gastric fundus through a diaphragmatic defect with evidence of incarceration (Figure 2). The cardinal feature of incarceration is the nasogastric decompression of the abdominal portion of the stomach in the presence of a dilated nondecompressed thoracic stomach. He underwent abdominal surgery within two hours of transfer. A necrotic gastric fundus was resected and the lateral defect in his diaphragm, a consequence of his prior surgery, was repaired with sutures. He is in good health with no sequelae one year postoperatively.

## 3. Case Two

A 67-year-old female presented to the emergency department with sudden-onset pleuritic chest pain thirteen months after laparoscopic left nephrectomy for a renal cell carcinoma. Of note, the patient had presented three weeks after undergoing nephrectomy with transient chest pain which resolved spontaneously. Examination and investigations at that time were normal.

**Figure 3:**
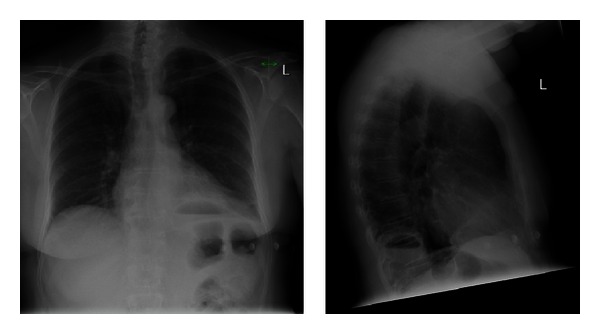
CXR Case 2; AP and lateral CXR demonstrating an air-fluid level in the posterior left hemithorax.

**Figure 4:**
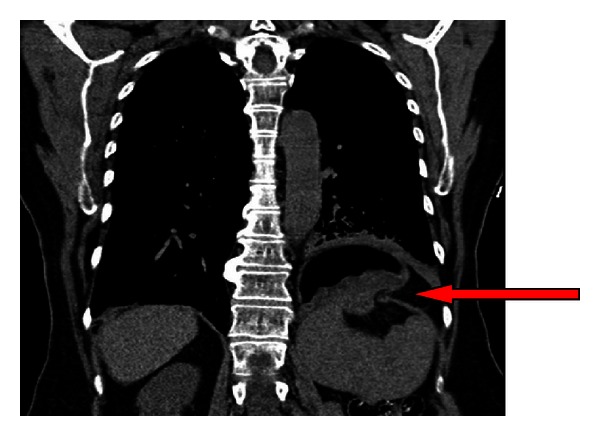
CT Case 2; coronal section demonstrating herniation of the gastric fundus through a diaphragmatic defect-note (as indicated by arrow) air-fluid level in herniated fundus indicating obstruction.

On presentation, a CXR (Figure 3) showed a new air-fluid level in the left hemithorax with poorly defined adjacent hemidiaphragm, suggestive of a lung abscess or possible diaphragmatic hernia. CT of the thorax and abdomen (Figure 4) revealed herniation of a portion of the body of the stomach through a 3.9 cm defect in the left hemidiaphragm. The presence of an air-fluid level indicated the likelihood of obstruction. 

**Figure 5:**
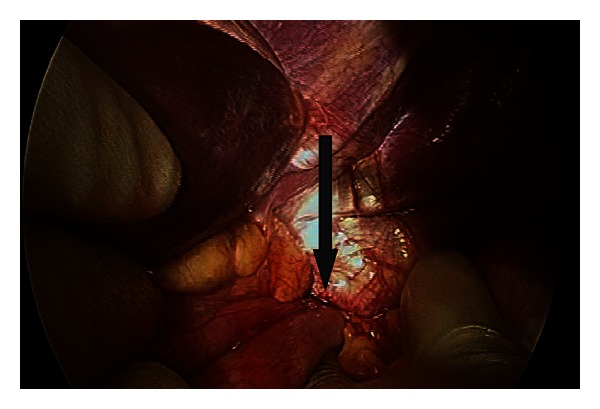
Operative photograph Case 2; herniation of the stomach through a diaphragmatic defect visualised at laparotomy.

The patient proceeded to surgery. A laparoscopy was performed and the fundus was visualised herniating through a narrow defect in the diaphragm (Figure 5). Reduction at laparoscopy was not possible necessitating an upper midline laparotomy. The diaphragmatic defect was enlarged anteriorly to allow reduction of the incarcerated gastric fundus. The diaphragmatic defect was visualised and repaired with interrupted sutures. The reduced gastric fundus showed no signs of necrosis. An on-table oesophagogastroduodenoscopy (OGD) confirmed that there was no necrosis but significant gastric hyperaemia. The patient had an uneventful postoperative course and was discharged home with followup.

## 4. Discussion

There is only one other case report in the literature describing an acute presentation of gastric perforation and diaphragmatic hernia after nephrectomy [10]. Liver, spleen, colon, and small bowel herniation have more commonly been described in diaphragmatic hernias after nephrectomy [5]. Iatrogenic diaphragmatic hernias may present with acute or chronic symptoms or may be found incidentally on follow-up imaging. Acute presentation of a diaphragmatic hernia varies depending on the extent and nature of the organ which has herniated. There may be epigastric or chest pain and dyspnoea due to pressure effect, or if stomach or bowel has become obstructed, the patient may vomit. Initial diagnosis can be challenging due to the nonspecific nature of the presenting symptoms, and often an acute myocardial infarction or a pulmonary embolus may be diagnosed. Delay in diagnosis poses a significant risk to the patient and a rapid deterioration can occur in the context of strangulation, as seen in Case 1. Careful attention to past surgical history may aid more rapid diagnosis [10].

Presentation of an iatrogenic diaphragmatic hernia can be delayed until months or years after the initial surgery [10], while cases have been reported where the defect has been identified and repaired intraoperatively during nephrectomy [13]. In both cases described here, presentation with a diaphragmatic hernia was followed between one and three years after the original surgery. It has been suggested that this delay in presentation is as a result of the gradual enlargement of small tears in the diaphragm not noticed at the time of surgery which develop over time with increases in intra-abdominal pressure associated with coughing and straining [14]. The withdrawal of positive pressure ventilation at the end of surgery, as well as subsequent relative increase in intra-abdominal pressure, has been a suggested mechanism for early presentation of a diaphragmatic hernia [13].

Diagnosis is typically established with plain radiograph or CT, although contrast studies, MRI, and ultrasound may also have a role [14]. Nasogastric tube insertion prior to imaging may help delineate a decompressed abdominal stomach from a dilated thoracic stomach, as in Case 1, strongly suggesting incarceration; however, a nasogastric tube could also perforate a necrotic gastric wall. 

Surgery is the treatment of choice either with direct suture repair, synthetic graft insertion, or a combination of both. Approach can be via laparotomy or thoracotomy. Transthoracic repair avoids the previous operative field in abdominal surgery patients [5, 7]. Transabdominal surgery is suggested in acute or unstable patients to allow better examination of intra-abdominal organs and dissection of adhesions. In both cases reported here, an abdominal approach was used, and one case highlights the diagnostic value of laparoscopy in selected cases and its potential for definitive treatment. Gastric necrosis must be resected, and careful examination of the entire stomach is needed once the hernia is reduced, with on-table endoscopy as used in Case 2 to aid examination. 

In conclusion, two recent cases at this centre describe the rare clinical entity of gastric strangulation or incarceration in the context of a diaphragmatic hernia following nephrectomy. A high index of suspicion is paramount, and general surgeons should be aware of the potential of this complication in patients presenting with atypical symptoms or critical illness after nephrectomy. If discovered on follow-up imaging, we would recommended that iatrogenic diaphragmatic hernias are repaired electively.
